# Implementing SARS-CoV-2 Testing during a Large-Scale Sporting Event in Africa: Lessons Learned from the Africa Football Cup of Nations Tournament in Cameroon

**DOI:** 10.4269/ajtmh.23-0898

**Published:** 2024-11-26

**Authors:** Boris K. Tchounga, Boris Tchakounte Youngui, Emilienne Epée, Tatiana Djikeussi, Joseph Fokam, André P. Goura, Loic Feuzeu, Muhamed Awulo Mbunka, Pallavi Dani, Shannon Viana, Anne Hoppe, Yap Boum, Rhoderick Machekano, Laura Guay, Anne-Cecile Zoung-Kanyi Bissek, John Ditekemena, Appolinaire Tiam, Alain G. Etoundi, Patrice Tchendjou, Michelle M. Gill

**Affiliations:** ^1^ Elizabeth Glaser Pediatric AIDS Foundation, Yaoundé, Cameroon, and Washington, District of Columbia;; ^2^ National Public Health Emergency Operations Coordination Centre, Ministry of Public Health, Yaoundé, Cameroon;; ^3^ Faculty of Medicine and Biomedical Sciences, University of Yaoundé, Yaoundé, Cameroon;; ^4^ Chantal BIYA International Reference Centre for Research on HIV/AIDS Prevention and Management, Messa, Yaoundé, Cameroon;; ^5^ Faculty of Health Sciences, University of Buea, Buea, Cameroon;; ^6^ FIND, Geneva, Switzerland;; ^7^ George Washington University, Milken Institute School of Public Health, Washington, District of Columbia;; ^8^ Division of Health Operational Research, Ministry of Public Health, Yaoundé, Cameroon;; ^9^ Department of Disease, Epidemic and Pandemic Control, Ministry of Public Health, Yaoundé, Cameroon

## Abstract

During the 33rd Africa Cup of Nations (AFCON) football tournament in Cameroon, organizers and health authorities required a negative SARS-CoV-2 test result <48 hours before entry and provided free SARS-CoV-2 testing and vaccination at stadium and fan zone entrances. We describe the outcomes and implementation of mandatory SARS-CoV-2 testing at fan zones during AFCON. All consenting fan zones attendees were administered an electronic questionnaire capturing exposure factors, COVID-19-like symptoms, and COVID-19 vaccination status, before being tested for SARS-CoV-2 using an antigen rapid diagnostic test (Ag-RDT). Participants testing positive were sampled for confirmatory real-time SARS-CoV-2 polymerase chain reaction (PCR) and sequencing for variant surveillance. The case detection rate was estimated using PCR-confirmed cases, and the challenges were summarized from staff discussions and project/study documentation. In total, 4,820 fan zone attendees (median [interquartile range] age 30 [24–38], 27.7% females) were tested for SARS-CoV-2, including 1,228 (25.5%) fully vaccinated. Of 4,820 participants, 148 (3.1%) had a positive Ag-RDT result, of whom 67 consented to PCR testing and 19 of 64 (29.7%) were confirmed PCR-positive. The case detection rate was 40.1 (95% CI: 24.2–62.7) per 10,000 attendees. The Omicron variant (B.1.1.529) was found in all 11 samples successfully sequenced. The implementation of mandatory SARS-CoV-2 Ag-RDT at fan zone entrances was challenged by high attendance volume just prior to matches, lobbying of economic stakeholders, and inconsistent quality assurance when using test kits. Despite the challenges encountered, implementing mandatory SARS-CoV-2 Ag-RDT at fan zones, was a unique opportunity for SARS-CoV-2 case identification and genomic surveillance.

## INTRODUCTION

The COVID-19 pandemic affected national health systems worldwide and was declared by the WHO as a global health threat.^1^^,^^2^ Since the onset of the COVID-19 pandemic in December 2019, the number of reported cases has reached approximately 771 million globally with more than 6.6 million related deaths as of October 27, 2023.^3^ In Cameroon, the epicenter of the epidemic in central Africa, the first two cases were confirmed on March 5, 2020, and as of October 8, 2023, 125,205 confirmed cases and 1,974 deaths had been recorded.^4^^–^^6^

The rapid spread of the virus worldwide was attributed to factors such as rapid mutations and high contagiousness of the new viral variants, especially Delta and Omicron, and to human factors such as travel and mass-gathering events (MGEs).^7^^,^^8^ In response to the pandemic, intensive awareness campaigns on preventive measures were implemented worldwide; face masks, hand sanitizers, polymerase chain reaction (PCR) test kits, and antigen rapid diagnostic test kits (Ag-RDTs) were rapidly developed and distributed, as well as new drugs and vaccines.^9^ In addition, population movement was limited through curfews, confinements, travel restrictions, and MGE that were all suspended or cancelled in 2020 because of the COVID-19 pandemic, including the Six Nations Rugby championship, the Mobile World Congress Barcelona, and the Umrah pilgrimage in Saudi Arabia.^9^^,^^10^

The Africa Cup of Nations (AFCON), which is the biggest and most popular sporting event on the African continent, was held in Cameroon in January and February 2022 in five cities (Yaoundé, Douala, Limbé/Buea, Garoua, and Baffoussam).^11^ Twenty-four country teams and their staff and officials were expected, and organizers anticipated more than 250,000 international visitors. Organizers considered the outcome of the Tokyo 2020 Summer Olympics, which were held without supporters and with epidemic control measures in place but were nonetheless followed by an increased transmission of SARS-CoV-2 in the general population, thus highlighting the risk of viruses spreading during sporting MGEs.^12^^,^^13^ As such, there were concerns about circulation of SARS-CoV-2 in the community and the risk of a new wave of infections at AFCON, particularly with the emergence of new SARS-CoV-2 variants, Delta and later Omicron, immediately before the event. Moreover, low vaccine coverage in Cameroon, estimated to 2.9% as of February 2022 called for more vigilance to contain the spread of SARS-CoV-2 during AFCON.^14^^–^^16^

The COVID-19 response in Cameroon was organized by the Ministry of Public Health (MoH) and implemented by the Public Health Emergency Operating Center with the objective of limiting disease transmission by prioritizing testing at airports and other country entry points, contact tracing, isolation of positive cases, social distancing, and personal hygiene.^17^^–^^19^ To mitigate the risk of a new wave of SARS-CoV-2 infections in Cameroon during or after AFCON, the Cameroon MoH, in partnership with the AFCON executive committee and the Ministry of Sports, decided to require proof of COVID-19 vaccination and negative SARS-CoV-2 test results to access stadiums, conference rooms, and official fan zones where supporters gather before, during, and after the matches.

After the implementation of MoH guidance at fan zones, we aimed to determine SARS-CoV-2 positivity rates and to identify the variants locally present during the AFCON competition; however, this was limited by the challenges experienced during implementation. We describe the experience of implementing mandatory SARS-CoV-2 Ag-RDT at entrances to fan zones, including testing results, challenges related to rapid testing for COVID-19 and laboratory processes, and lessons learned during the 33rd AFCON in Cameroon.

## MATERIALS AND METHODS

### Study design and settings.

A cross-sectional study was conducted during the 33rd AFCON tournament played in Cameroon from January 9 to February 6, 2022. The study took place in Yaoundé and Douala, Cameroon’s political and economic capitals respectively. These are the two biggest cities in the country, with the highest reported SARS-CoV-2 prevalence, where the opening ceremony, semifinal, and final AFCON matches took place. In each city, the study was implemented in official AFCON fan zones, which are open spaces that can accommodate between 3,000 and 5,000 fans and that telecast matches and offer drinks, food, and goods for sale.

### SARS-CoV-2 control policy during the 33rd AFCON.

Before AFCON, the Cameroon MoH, in agreement with the AFCON executive committee, issued guidance for COVID-19 prevention and control during the tournament. This guidance required that any supporter who wanted to access stadiums and fan zones be fully vaccinated and present a negative SARS-CoV-2 Ag-RDT or PCR test taken less than 48 hours before each entry. A quick response (QR) code with a “health pass” was issued to any person meeting the two requirements at each entry of the fan zone. A checkpoint was set at the entrance to each fan zone, where two MoH agents and one police officer were posted to verify the health pass by scanning the QR code. SARS-CoV-2 testing and vaccination could also be obtained at health facilities in all the health districts, and a health pass issued for documentation. Participants without a valid (issued within past 48 hours) health pass were to be denied access and referred to one of the SARS-CoV-2 testing and vaccination points around the stadium or fan zone to obtain valid documentation before entry.

### SARS-CoV-2 testing at fan zone entrances.

At the entrance to each fan zone, a temporary health post was installed, made up of four units: a SARS-CoV-2 testing unit in charge of performing sample collection and testing, a COVID-19 immunization unit in charge of vaccination, a water sanitation and hygiene unit in charge of infection prevention and control, and a documentation unit in charge of visitor registration, and verification and issuance of the health pass. Individuals presenting at the post to obtain a health pass were offered SARS-CoV-2 testing and vaccination by trained MoH healthcare workers and administered per national and manufacturer guidelines using nationally approved test kits and vaccines. Those testing negative for SARS-CoV-2 and who were vaccinated were issued a valid health pass to access the stadium and fan zones, and those testing positive were referred to a care and treatment team for posttest counseling and follow-up per national treatment guidelines.

### Study participants and enrollment.

Research assistants were embedded into MoH teams at health posts installed at the two largest venues in Yaoundé and Douala and were performing study procedures integrated with SARS-CoV-2 testing services. All fan zone attendees, aged >2 years or older (manufacturer age limit for test kit diagnostic accuracy), presenting at the health post to request a health pass were offered free-of-charge SARS-CoV-2 Ag-RDT testing, and administered an electronic questionnaire capturing demographics, COVID-19-like symptoms, SARS-CoV-2 exposure, existing comorbidities, and COVID-19 immunization status after a verbal consent. Participants aged 21 years or older who tested positive were asked to sign a written consent for the genomic surveillance survey and provided a second nasopharyngeal sample for PCR testing and whole genome sequencing (WGS), to be performed in the virology unit of the Chantal Biya International Research Center. The PCR test and WGS results were sent to the research team using sample referral forms.

### SARS-CoV-2 testing and laboratory procedures.

SARS-CoV-2 Ag-RDT was performed using the PANBIO™ COVID-19 Ag Rapid Test Device (Abbott Rapid Diagnostic International limited, Ballybrit Ireland), a rapid chromatographic immunoassay for the qualitative detection of SARS-CoV-2 nucleocapsid antigen present in human nasopharyngeal samples, endorsed by WHO and authorized by the Cameroon MoH. The SARS-CoV-2 reverse transcription (RT)-PCR was performed using the DaAn gene assay (Guangzhou, China), following the manufacturer’s instructions. The RT-PCR amplification was conducted using QuantStudio qPCR Systems (Thermo Fisher Scientific, Waltham, MA). The protocol used probes targeting the open reading frame (ORF) gene and the nucleocapsid (N) protein gene, with a lower limit of detection of 500 copies/mL and an amplification reaction of 45 cycles. Samples were considered positive for SARS-CoV-2 for CT values <37 amplification cycles. The amplification and sequencing procedure were detailed elsewhere.^20^ Sequences were aligned, assembled, and edited by the reference sequence using Seqscape V2.7. Spike sequences were interpreted using the COV19 Stanford algorithm (https://covdb.stanford.edu).

### Audit procedures.

The audit procedure was planned to take place in case any challenge was observed with the testing procedure or results at the health post of the fan zone (SARS-CoV-2 Ag-RDT) or any incident or discordant results reported at the referral laboratory of the study. The country-approved manual of procedures for the AFCON health surveillance provided a checklist for the assessment of SARS-CoV-2 Ag-RDT testing points, that was used during the audit at fan zones. This checklist assessed three main domains: 1) SARS-CoV-2 Ag-RDT testing flow (installation, registration, sample collection, testing, result reading and announcement, registration and issuance of health pass); 2) personnel and technical skills (number of trained personnel, experience on SARS-CoV-2 testing, training on test kit and utilization, mastering of manufacturer standard operating procedures [SOPs] for each specific test kits); 3) test kits and sample management (sample collection conditions, test kit storage, temperature monitoring, sample transportation system for genomic surveillance).

### Monitoring of study challenges.

Early in the course of study implementation, unexpected challenges were reported by the coordination team, and a close monitoring of the study procedures took place, led by sponsor, donors, and investigators, to understand and document, the challenges affecting the study procedures. This close monitoring consisted of biweekly calls for situational update and review of the coordination and monitoring documents produced during the implementation phase of the study (minutes of coordination meetings, monitoring reports, enrollment logs, issue logs). In addition, data review meetings were organized to share the progress and challenges with MoH, and a final report of the project was prepared and communicated to donors. At the end of the study, the following documents were reviewed and analyzed to describe the challenges with their causes and consequences, and the mitigation actions: minutes of weekly coordination meeting (12), minutes of touch base call with the donor (10), weekly monitoring reports from both regions (16), issue logs from each region (2), reports of the supervision by regional delegation of public health in each region (4), report of the audit of SARS-CoV-2 testing process at the Douala fan zone (1), and final study report (1). In addition, documents such as service notes from mayors, governors, and health authorities (8); circular letters from MoH (2); and newspaper report of interviews with government officials were also reviewed to triangulate information reported in the study documents.

### Data collection and analysis.

We described the characteristics of participants testing for SARS-Cov-2 Ag RDT, including the distribution of comorbidities and COVID-19-like symptoms at the time of testing. We calculated the overall SARS-CoV-2 case detection rate as the number of SARS-CoV-2 PCR positive tests divided by the total number of tests conducted, expressed per 10,000 tests. Assuming a Poisson distribution for the number of positive tests, we estimated the associated precision as the 95% CI of the case detection rate. Frequencies and proportions of SARS-CoV-2 variants were estimated among SARS-CoV-2-positive individuals. Implementation challenges and mitigation actions were identified and abstracted from various source documents available during the project and cross-checked with MoH and other publicly available government documents and media announcements. These challenges were grouped following a thematic approach and summarized in tables with causes consequences and mitigation actions.

## RESULTS

### Characteristics of the population tested at the fan zone health posts.

**Figure 1.**
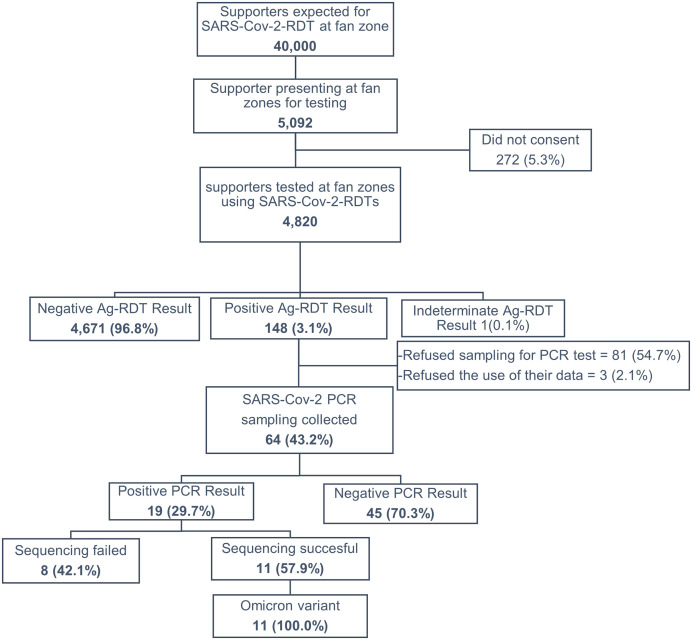
Flow chart of fan zone attendees tested during the 33rd Africa Cup of Nations tournament in Cameroon in 2022. Present results of SARS-CoV-2 testing among individuals at fan zones. Those with positive antigen rapid diagnostic test results show those accepting or refusing further polymerase chain reaction (PCR) testing. Those who agreed to be tested indicate the results of PCR testing and the variant identified for those with successful sequencing.

**Figure 2.**
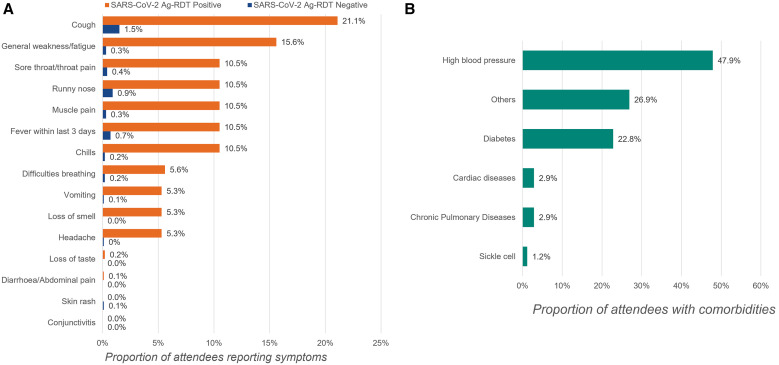
Portion of attendees (**A**) with symptoms and (**B**) reporting comorbidities.

A total of 5,092 people came at the study’s fan zone health posts to seek SARS-CoV-2 Ag-RDT, of whom 4,820 (94.6%) were tested and enrolled in the study (Figure 1). The 272 who did not provide verbal consent to be enrolled in the study were also offered SARS-CoV-2 testing services, but their data were not included in the study database. Among the 4,820 participants enrolled in the study, 2,503 (51.9%) were in Douala and 2,317 (48.1%) in Yaoundé; the median age was 30 (interquartile range: 24–38) years, 3,487 (72.3%) were males, and 4,657 (96.6%) reported living in Cameroon (Table 1). The reasons for visiting the fan zone health post among the 4,820 participants were the need for the health pass to access football matches in fan zones for 3,812 (79.1%) and the need for the health pass for other reasons, including travel, for 1,008 (20.9%). Of the 4,820 participants, 180 (3.7%) reported the presence of at least one COVID-19-like symptom, and 171 (3.6%) reported at least one comorbidity; 476 (9.9%) reported having tested positive for SARS-CoV-2 in the past, 461 (96.9%) of whom were declared cured. COVID-19-like symptoms such as cough, general weakness/fatigue, sore throat/throat pain, runny nose, muscle pain, fever, and chills were more frequent in attendees with a positive SARS-CoV-2 Ag-RDT result (Figure 2A). Nearly half of attendees who received SARS-CoV-2 testing reported having high blood pressure with approximately one-quarter reporting diabetes and other comorbidities (Figure 2B). Among the 4,820 participants, 1,228 (25.5%) reported being fully vaccinated, and the single-dose Johnson & Johnson COVID-19 vaccine was the vaccine received by most, accounting for 888 of 1,228 (69.0%) of those fully vaccinated (Table 1).

**Table 1:** Demographics, clinical characteristics, and COVID-19 history among people tested for SARS-CoV-2 at fan zone entrances during the 33rd Africa Cup of Nations in Cameroon

Characteristics	Total (*N* = 4,820) *n* (%)	Douala (*n* = 2,503; 51.9%) *n* (%)	Yaoundé (*n* = 2,317; 48.1%) *n* (%)
Age, median [interquartile range], years	30 [24–38]	28 [23–35]	32 [24–41]
Sex			
Male	3,487 (72.3)	1,891 (75.6)	1,596 (68.9)
Female	1,333 (27.7)	612 (24.4)	721 (31.1)
Country of residence			
Cameroon	4,657 (96.6)	2,436 (97.3)	2,221 (95.6)
Other African	55 (1.2)	11 (0.5)	44 (1.9)
Non-African	108 (2.2)	56 (2.2)	52 (2.2)
Availability of health pass			
No health pass	3,615 (75.0)	1,914 (76.5)	1,701 (73.4)
Invalid health pass	1,205 (25.0)	589 (23.5)	616 (26.6)
Reason for visiting fan zone			
Watching football match	1,139 (23.6)	174 (6.9)	965 (41.6)
Referred from stadium	2,673 (55.5)	1,876 (74.9)	797 (34.4)
Renew health pass/other	1,008 (20.9)	453 (18.1)	555 (23.9)
Presence of symptoms			
≥1	180 (3.7)	34 (1.4)	146 (6.3)
None	4,640 (96.3)	2,469 (98.6)	2,171 (93.7)
Presence of comorbidities			
≥1	171 (3.6)	48 (1.9)	123 (5.3)
None	4,649 (96.4)	2,455 (98.1)	2,194 (94.7)
History of COVID-19 disease			
Tested positive at least once	476 (9.9)	242 (9.7)	234 (10.1)
None	4,344 (90.1)	2,261 (90.3)	2,083 (89.9)
Outcome previous COVID (*n* = 476)			
Declared cured	461 (96.9)	233 (96.3)	228 (97.4)
Not declared cured	15 (3.1)	9 (3.7)	6 (2.6)
COVID vaccine status			
Fully vaccinated	1,228 (25.5)	547 (21.8)	681 (29.4)
Not/partially vaccinated	3,592 (74.5)	1,956 (78.2)	1,636 (70.6)
Type of vaccine received			
AstraZeneca	109 (8.5)	39 (6.9)	70 (9.7)
Sinopharm	112 (8.7)	40 (7.1)	72 (10.0)
Johnson & Johnson	888 (69.0)	423 (74.6)	465 (64.7)
Pfizer	141 (11.0)	53 (9.3)	88 (12.3)
Moderna	23 (1.8)	7 (1.2)	16 (2.2)
Other	13 (1.0)	5 (0.9)	8 (1.1)

### Testing cascade and SARS-CoV-2 case detection rate.

Among the 4,820 participants who provided samples for SARS-CoV-2 Ag-RDT, 148 (3.1%) tested positive and were asked to provide a second sample for PCR test confirmation. Of the 148 participants with a positive Ag-RDT test result, 64 (43.2%) provided samples for PCR testing, and 19/64 (29.7%) were confirmed positive for SARS-CoV-2 (Figure 1). Sequencing results were obtained for 11/19 (57.9%) samples and all were the Omicron variant B.1.1.529 (Figure 1). The estimated case detection rate was 40.1 cases per 10,000 persons tested (95% CI: 24.2–62.7 cases per 10,000 persons tested).

### Challenges during implementation of SARS-CoV-2 testing at fan zones.

#### Challenges maintaining the SARS-CoV-2 testing mandatory at fan zones.

The first challenge was identified when analyzing the unexpected low number of people tested at fan zones during AFCON. According to the AFCON fan zone managers, during the tournament, attendance in each fan zone varied from a minimum of 500 fans (on days when no match was played or nonpopular teams were playing) to a maximum of 3,500 to 4,000 fans (on days the Cameroon team or other popular teams were playing). We estimated that approximately 45,000 (40,000–50,000) fans attended each study fan zone during the 29 days of the AFCON period, and considering that each participant with a negative SARS-CoV-2 test was requested to renew the test every 48 hours as per the MoH recommendation, we could expect to test no less than 20,000 fans in each fan zone if the valid health pass was mandatory to enter the fan zone all the time. However, during the tournament, only 5,092 of 40,000 expected (12.7%) fans showed up at the health post seeking a SARS-CoV-2 test. The analysis of study documents revealed that two operational challenges were behind this low number of people tested at fan zone. First, 1 hour before the beginning of the matches, the majority of fan zone attendees started crowding at the entrance of fan zones, and when denied access (due to an invalid health pass), they were referred to the health post where two registration units, two testing units, one result reading and validation unit, and one immunization unit, each staffed by healthcare workers, were to provide SARS-CoV-2 testing services. Each participant was spending 5–10 minutes for registration (including symptoms and risk factors screening), 5–10 minutes for testing (preparation with infection prevention control measures, sample collection and dropping on the kit), 15 minutes for result reading, and 5 minutes for result recording and health pass issuance, for a total of 30–40 minutes before getting a valid health pass to enter the fan zone. This long waiting time close to the beginning of football matches disappointed many attendees who either became aggressive and requested immediate access to the fan zone or decided to watch the match elsewhere.

The second operational challenge was the pressure from economic stakeholders (e.g., vendors, businesses) in the fan zones, whose interest was to fill the fan zones with attendees. During the first days of the tournament, the strict control of the health pass at fan zone entrance, combined with the disappointment of supporters who could not get tested on time or did not want to get tested and preferred watching the match elsewhere, led to lower attendees in the fan zones, affecting the incomes of economic stakeholders. To overcome this challenge, the businessowners in the fan zone and the fan zone managers started lobbying against the health pass policy at fan zones. As a result of these two challenges, the control of the health pass at fan zone entrances was relaxed and sometimes removed (especially for the days popular teams were playing) to permit all supporters who agreed to wear a face mask and sanitize their hands into fan zone. Therefore, SARS-CoV-2 testing at fan zones, despite being officially mandatory, became voluntary after the second week of the tournament, making it impossible to test all those eligible for testing.

#### Challenge with quality assurance of SARS-CoV-2 Ag-RDT use in fan zones.

The second challenge identified was the unexpected high proportion of discordance between SARS-CoV-2 Ag-RDT and PCR results in the Douala Fan zone. Of the 64 PCR samples received at the laboratory, 56 came from Douala fan zone and only 11 (19.6%) of them were confirmed positive for SARS-CoV-2 with RT-PCR test, whereas 100% of the eight samples coming from the Yaoundé fan zone were confirmed positive. The main findings of the laboratory audit are summarized in Tables 2 and 3. At the Douala fan zone health post, the audit revealed appropriate staffing (32 trained staff) and dedicated setting for each unit (e.g., triage, registration) to implement project activities. However, despite the lack of temperature monitoring, there was evidence suggesting low to medium risk for the testing material to have been exposed to temperature >30°C during the implementation phase (Table 2).

**Table 2:** Finding of the audit performed on the SRAS-CoV-2 Ag-RDT flow at the Littoral fan zone’s testing point discrepancies between reverse transcription polymerase chain reaction and Ag-RDT results

Domain	Item Assessed	Method, Mean, Source of Verification	Summary of Findings	Conclusion
Testing capacities, logistics, and biosafety at the fan zone	Personnel (quality and quantity)	Checking the listing and attendance registers	32 trained staff: 5 senior laboratory technicians, 4 laboratory technicians, 4 assistant laboratory technicians, 7 clinical doctors, 2 pharmacists, and 10 assistants for WASH	Appropriate number of staff by cadre available and trained for SARS-CoV-2 Ag-RDT testing
		One-on-one and group discussions with manager and staff	Regular trainings on testing, case management, drug and vaccine delivery, stock management, and WASH according to each cadre	
	Settings, logistics and flow	Monitoring visit to fan zone testing points during busy periods	Dedicated places for 1) triage, 2) registration, 3) sample collection and testing, 4) reading and result validation, 5) negative result announcement, 6) positive result announcement and medical interview, 7) vaccine administration	Testing points well organized to avoid confusion and ease the work flow
			All these specific places were under tents installed and organized to ease the testing flow	Low/medium risk of testing material and personnel exposed to temperature (Temp. >30°)
			Absence of air conditioner under the tent, leading to ambient temperatures above 30°C	
	Security and biosafety	Monitoring visit to fan zone testing points during busy periods	Availability of protective equipment for personnel and participants (face mask, gloves, overcoat, hand sanitizers) and safety box	No evidence of biosafety challenge or risk for contamination
		Group discussion with the staff	WASH team available with material and strict on the timing for sanitization	
Personnel’s practices with SARS-CoV-2 Ag-RDT	Checking batch number and expiry date	Group discussion with the testing staff	Systematically done by the team but not documented in a register as recommended “Abbott *Pan* bio” with batch (41ADG627B) was recognized as the only test kit used for the study	No/minimal risk of using expired test kit with this batch number of “Abbott *Pan* bio”
	Performing quality control on each box before starting using the kits		This was not systematically done by the team and also not documented when done	Medium/considerable risk of using invalid test kits that should be removed from the stock
			Unexpected results during quality control observed with two boxes (negative control showed positive results), but this was not documented and the team unable to provide these boxes	
	Mastering manufacturer SOP for the use of the test kit		No job aid displayed or easily accessible to remind the staff on the procedure	Low/medium risk of invalid results failure to follow manufacturer (pan bio) SOPs when using the test kit
			Discrepancies in the answers about the number of drops necessary during the test (3 for some staff instead of 5 as recommended) Answers on the knowledge consistent with answers on the practices	
			Lack of chronometers at the reading and validation point, and variability in reading time (15 minutes or less for some staff and 20 minutes for others)	
			Use of the buffer fluid from one box to perform test with kits from another box	

Ag-RDT = antigen rapid diagnostic test; SOP = standard operating procedure; WASH = water, sanitation and hygiene.

**Table 3:** Findings of the audit performed on the SARS-CoV-2 PCR testing flow, from sampling at fan zone to testing in the laboratory and results dissemination, discrepancies between RT-PCR and antigen rapid diagnostic test results

Item Assessed	Method, Mean, Source of Verification	Summary of Findings	Conclusion
Sample collection; cold chain and transportation of samples	Visit of the testing points to check availability and functionality of the cold chain	Availability of a refrigerator on site (4–8°C)	No evidence of issue with sample collection and transportation to the laboratory
		Availability of cooler boxes with dry ice	
		Availability of appropriate packaging for biohazard transportation	
	Group discussion with the staff	Use of different viral transport medium for PCR sample but all were provided by the Ministry of Health	
Validity of biological samples received	Checklist of sample control and receipt in the laboratory	All the 67 samples were declared valid upon arrival and accepted by the team	No evidence of issue or very marginal issue with quality of samples tested for RT-PCR at the laboratory
	Internal sample quality control available in the PCR machine for validation of sample quality	66 samples validated by the internal quality control of the PCR machine	
		1 sample with very low signal during internal quality control not validated	
Conformity of PCR test performed	Laboratory journal (systematic realization of positive and negative quality control tests on each batch and conformity of the results)	For each series of RT-PCR tests done, all the positive and negative controls have been performed and the results were in favor of a valid test	No evidence of contamination during the manipulation process All results validated by the internal quality controls
Concordance of results sent back	Cross matching the code of samples transmitted from the fan zone and the code of results transmitted by the laboratory to ensure appropriate result was given to the right person	All the codes from the fan zones and those from the laboratory matched with the study ID of each participant.	No evidence of mix-up or confusion in the results returned
		There were no duplicates, no missing IDs and no results with an unknown participant	

RT-PCR = reverse transcription-polymerase chain reaction.

Analyzing the personnel answers about SARS-CoV-2 testing, the audit found evidence that the team used at least two boxes of test kits that failed quality control (negative control displaying positive result) and also that the team sometimes failed to follow the manufacturer SOPs for the use of “pan bio” test kits (Table 2). The audit of the RT-PCR flow from Douala fan zone to the laboratory and the testing process until result dissemination did not find any evidence of issues with the sample transportation system and sample validity, laboratory quality control, contamination during the test process, and result dissemination (Table 3). The audit report concluded that the discrepancies observed between SARS-CoV-2 Ag-RDT and RT-PCR tests were probably due to operational challenges such as high temperature (>30°C) that could have affect the functioning of test kit and failure from the testing team to follow manufacturer’s SOPs when using test kits.

The last challenge we observed during the implementation of the study was the high proportion of participants tested positive for SARS-CoV-2 Ag-RDT who refused to provide a second sample for RT-PCR confirmation. Analyzing study enrolment logs and issue logs completed by research assistants, refusal reasons were available in some cases and included “lack of trust in the results” and “need for time to consider and discuss with family.

## DISCUSSION

This study is among the first reports on SARS-CoV-2 testing in fan zones during sporting MGEs in Africa. People tested at fan zones were mainly 30-year-old men, living in Cameroon, who were coming to watch football matches in fan zones; one-fourth were fully vaccinated, and few presented COVID-19-like symptoms and comorbidities. During AFCON, the estimated case detection rate at fan zones was 40.1 cases per 10,000 persons tested and Omicron (B.1.1.529) was found in all successfully sequenced samples. During the AFCON tournament, the main challenges encountered were difficulty maintaining SARS-CoV-2 testing mandatory for those with invalid health pass at fan zone entrance, difficulty ensuring quality assurance during SARS-CoV-2 Ag-RDT, and reluctance of participants to provide second PCR sample.

### Opportunity to implement testing at fan zone entrances.

Fan zones were created 20 years ago and remain a key method of accommodating patrons who are unable to access stadiums during large-scale sporting events, such as international football tournaments, as well as those who want to enjoy pre- and post-match events outside of stadiums.^21^ The rapid development and growth of fan zones has made them an opportune location for security and public health interventions, especially during epidemics. In our study, SARS-CoV-2 Ag-RDT offered free of charge at fan zone entrances enabled the detection of 40.1 cases per 10,000 persons tested. Although the case detection rate was low, the implementation of testing at fan zone entrances allowed for the early detection of symptomatic and asymptomatic SARS-CoV-2 infection and for the offer of appropriate care for participants testing positive to protect their health and prevent the spread of SARS-CoV-2.

Thus, offering free SARS-CoV-2 testing at entrances to MGEs could be an important public health measure to increase SARS-CoV-2 detection.^22^^–^^30^ Our study also revealed that the Omicron variant (B.1.1.529) was the only variant circulating among participants who tested positive for SARS-CoV-2 during AFCON, confirming the rapid spread of Omicron and its surpassing the spread of the Delta variant, as suggested by country epidemiologic reports of that period.^31^ Hence, implementing free SARS-CoV-2 testing at fan zone entrances during large-scale sporting or social MGEs is a critical opportunity for reinforcing epidemic and genomic surveillance of emerging and rapidly evolving public health threats such as SARS-CoV-2.^32^^–^^36^ Testing at fan zones should be free of charge and mandatory for all fan zone attendees to achieve the expected public health impact.^37^

### Challenges implementing mandatory testing at fan zone.

Our experience of implementing SARS-CoV-2 testing mandatory at fan zone entrances was not successful and highlights some important challenges that need to be anticipated and mitigated before a mass gathering event. First, it is important to anticipate that majority of attendees will come less that 1 hour before the beginning of the event and that all those who need to get tested will not accept waiting for a long time and risking missing the event they came to see. In our study, despite an appropriate number of trained staff and physical space allocated to the health post for SARS-CoV-2 testing, it was challenging to satisfy demand once many people had gathered at the entrance. One solution to address this could have been to reorganize the teams to have more people working 2 hours before and 2 hours after the match, instead of having half of the team working full time one day and the other half working full-time the next day, as we did. There is a need for different approaches aligned with available human and financial resources to address the flow of fans coming immediately before the event and needing to get tested in a very short period of time. Large-scale communication and awareness campaigns through various media platforms, as well as presence of police at entrance of the fan zones to deny access to those not complying with health pass policy, can certainly help, but the main challenge remained the management of a large number of fans in a short period of time.

In our study, another important challenge was that fans who were unwilling or unable to get tested on time were denied access to fan zones, and they moved to another place with less restrictions than the fan zone. Knowing that a fan zone is primarily a business, economic stakeholders who invested in these fan zones lobbied to remove the requirement for the health pass at fan zones, and the SARS-CoV-2 testing became more voluntary than mandatory. Thus, there is a need to identify strategies and approaches to help enforce mandatory testing at fan zone entrances without conflicting with the interests of fan zone stakeholders. Possible solutions to ensure the success of similar testing efforts can include involving economic stakeholders in planning and discussions and proposing promotional gifts or discount codes to those tested at the fan zone during peak hours, including agreements in their contracts for compliance to public health measures set by the government.

We also identified lack of quality assurance during the use of SARS-CoV-2 Ag-RDT at fan zones due to human resource and logistical challenges and to working conditions that usually do not occur in routine testing settings. Specific quality assurance process should be designed and implemented when planning for SARS-CoV-2 Ag-RDT during large-scale MGEs. In addition, systematic refresher trainings should be organized for the team working during MGEs, and job aids should be available and displayed at the testing site to avoid mistakes due to fatigue or overconfidence. Finally, organization of testing during MGEs required strict monitoring of temperature for the storage of test kits and other testing material. In our study, we found the testing material was not stored or used at the optimal temperature recommended by the manufacturer, and this could have affected the quality of the results. Thus, planning for testing during MGEs should take into account the temperature of the working atmosphere and the storage of testing material to avoid possible concerns with the results.

### Study limitations.

One of the initial objectives of this study was to report on the prevalence of SARS-CoV-2 among supporters attending fan zones during AFCON. As such, it was important to test all the attendees presenting at fan zones without a valid health pass. Because of the operational challenges, we were not able to estimate population-based prevalence of SARS-CoV-2 because we could not test or otherwise determine status of all supporters at study fan zones. We planned to conduct genomic surveillance by performing WGS in all the participants testing positive for SARS-CoV-2 Ag-RDT. Unfortunately, we experienced a low acceptance rate for the PCR sampling, mainly because of mistrust in their Ag-RDT result, which reduced the proportion of positive samples with genome sequencing. However, we were able to perform the genome sequencing for those who provided the PCR sample and identified Omicron as the only circulating variant during the AFCON, as had already been shown in national data.^20^ We noticed a high discordance rate between antigen testing and positive PCR results, limiting the number of positive PCR being sequenced. This type of discordance is not rare and can be expected in some circumstances, but in our study, the proportion of discordance was high, leading the team to conduct an audit in the Douala Fan zone.^38^ However, we were able to report on the case detection rate based on PCR results among all participants tested at fan zones, which is a good reflection of the epidemic trends among the population interested in football matches. In addition, this study described the operational and quality assurance challenges that should be anticipated when planning for SARS-CoV-2 testing or other disease testing during AFCON tournaments and for future large-scale sporting events occurring during epidemics or pandemics.

## CONCLUSION

Fan zones, like other entry points to MGEs, can be used as platforms for epidemic and genomic surveillance during large-scale MGEs, but not without challenges. Implementing mandatory SARS-CoV-2 Ag-RDT at the entrances to fan zones during a sporting MGE was met with reluctance from attendees and economic stakeholders and faced insufficient laboratory quality assurance. As such, the implementation of mass testing during large events should be planned carefully, involving stakeholders early in the planning process, including those with an economic interest and attendees or other beneficiaries, to help identify appropriate mitigation actions for anticipated challenges and to ensure a commitment to and respect of any preventive measures adopted for public health interest. Laboratory quality assurance is critical when implementing mass testing, including during large-scale sporting events and should be strengthened at all testing points.
